# Developing a Patient Profile for the Detection of Cognitive Decline in Subjective Memory Complaint Patients: A Scoping Review and Cross-Sectional Study in Community Pharmacy

**DOI:** 10.3390/healthcare13141693

**Published:** 2025-07-14

**Authors:** María Gil-Peinado, Francisco Javier Muñoz-Almaraz, Hernán Ramos, José Sendra-Lillo, Lucrecia Moreno

**Affiliations:** 1 Cátedra DeCo MICOF-CEU UCH, Universidad Cardenal Herrera-CEU, 46115 Valencia, Spain; maria.gilpeinado@alumnos.uchceu.es (M.G.-P.); malmaraz@uchceu.es (F.J.M.-A.); ramgarher@alumnos.uchceu.es (H.R.); j.sendralillo@gmail.com (J.S.-L.); 2 Muy Ilustre Colegio Oficial de Farmacéuticos de Valencia, 46003 Valencia, Spain; 3 Department of Mathematics, Physics and Technological Science, Universidad Cardenal Herrera-CEU, CEU Universities, 46115 Valencia, Spain; 4 Department of Pharmacy, Universidad Cardenal Herrera-CEU, CEU Universities, 46115 Valencia, Spain

**Keywords:** cognitive dysfunction, screening, internet, social media, sleep, education, preventive services, primary health care

## Abstract

Background and Objectives: Early detection of cognitive decline (CD) is crucial for managing dementia risk factors and preventing disease progression. This study pursues two main objectives: (1) to review existing cognitive screening practices implemented in community pharmacy settings and (2) to characterize the cognitive profile of individuals eligible for screening in this context. Materials and Methods: This study was conducted in two phases. First, a scoping review of cognitive screening tools used in community pharmacies was carried out following PRISMA-ScR guidelines. Second, a cross-sectional study was performed to design and implement a CD screening protocol, assessing cognitive function. Data collection included demographic and clinical variables commonly associated with dementia risk. Decision tree analysis was applied to identify key variables contributing to the cognitive profile of patients eligible for screening. Results: The scoping review revealed that screening approaches differed by country and population, with limited pharmacy involvement suggesting implementation barriers. Cognitive screening was conducted in 18 pharmacies in Valencia, Spain (1.45%), involving 286 regular users reporting Subjective Memory Complaints (SMC). The average age of participants was 71 years, and 74.8% were women. According to the unbiased Gini impurity index, the most relevant predictors of CD—based on the corrected mean decrease in corrected impurity (MDcI), a bias-adjusted measure of variable importance—were age (MDcI: 2.60), internet and social media use (MDcI: 2.43), sleep patterns (MDcI: 1.83), and educational attainment (MDcI: 0.96). Simple decision trees can reduce the need for full screening by 53.6% while maintaining an average sensitivity of 0.707. These factors are essential for defining the profile of individuals who would benefit most from CD screening services. Conclusions: Community pharmacy-based detection of CD shows potential, though its implementation remains limited by issues of consistency and feasibility. Enhancing early dementia detection in primary care settings may be achieved by prioritizing individuals with limited internet and social media use, irregular sleep patterns, and lower education levels. Targeting these groups could significantly improve the effectiveness of CD screening programs.

## 1. Introduction

Approximately half of older adults report memory complaints without experiencing functional decline, a condition known as Subjective Memory Complaint (SMC) [1]. SMC refers to self-perceived memory difficulties that are not confirmed through formal assessments but may indicate early-stage dementia [2]. SMC has been associated with a twofold increase in the likelihood of developing dementia [3] and may be an early symptom of Alzheimer’s Disease (AD) in some patients [4]. Additionally, SMC may appear at stage 2 of AD according to the NIA-AA criteria, which represents the stage preceding mild cognitive impairment (MCI) [5].

MCI is an intermediate stage between age-related cognitive dysfunction and more severe dementia. It can encompass difficulties with memory, language, thinking, and judgment that are more pronounced than the typical changes associated with aging [6]. The belief that memory decline is a normal aspect of aging often contributes to delays in detection [7]. Nevertheless, since the characterization of MCI as a clinical condition, there has been significant interest in identifying affected individuals as early as feasible. In fact, several studies have assessed the therapeutic potential and economic benefits of early diagnosis within the healthcare system [8]. Given its subjective nature, accurately assessing MCI requires scientifically validated psychometric tests [9].

Subjective Cognitive Decline (SCD) describes worsening cognitive perception in cognitively normal older individuals, potentially indicating underlying neuronal damage preceding Objective Cognitive Decline (OCD). Research indicates that individuals with SCD have an increased risk of developing MCI and a twofold risk of dementia [10]. Therefore, a patient’s subjective perception alone may be sufficient to warrant further testing [11].

Since age is the primary risk factor for dementia and no definitive treatment is currently available, its progression is often considered inevitable [12]. However, research suggests that modifiable lifestyle factors influence dementia development, supporting the potential for prevention through effective public health strategies [13]. Early detection of dementia offers significant benefits, including timely initiation of medication to control symptoms, financial planning, participation in support groups, and lifestyle modifications, ultimately reducing economic impact and enhancing quality of life for patients and caregivers [14]. In Spain, cognitive screening tests are widely utilized in primary care [15].

Dementia detection is not straightforward, and without objective markers, the diagnosis and treatment are often delayed. Through this screening, SCD is objectivized. However, it cannot be definitively determined whether the patient suffers from MCI or not. Referral to a neurologist is necessary to assess the need for further checks and to issue a diagnosis that confirms this suspicion. The screening results suggest cognitive impairment, but a diagnosis cannot be made based solely on the questionnaires because the diagnosis of AD can involve a combination of detailed medical history, physical examination, laboratory testing, cognitive assessments, and brain imaging scans conducted by a physician [14].

As the first point of access to the healthcare system, Spanish community pharmacies have the potential to play a crucial role in identifying early signs of cognitive decline (CD) and facilitating timely referrals to primary or specialized care. Community pharmacies are healthcare establishments that are conveniently located, easily accessible, and trusted by patients. Pharmacists, with their training and experience, are actively involved in providing healthcare and improving patients’ quality of life, including disease prevention [6,7]. In fact, community pharmacies often serve as patients’ initial point of contact with the healthcare system [16]. This makes pharmacists well-suited healthcare professionals to provide Pharmaceutical Care Services, such as CD screening. Additionally, they also play a crucial role in providing counselling and health education, which are essential in managing modifiable factors associated with the development of dementia [6,7]. However, evidence on the effectiveness and feasibility of cognitive screening in this setting remains limited and heterogeneous.

To design an effective CD screening strategy, the application of machine learning tools has become essential. The selection of these techniques requires balancing model interpretability and performance. While methods such as random forest or artificial neural networks exhibit strong predictive performance, they lack interpretability. Conversely, stepwise logistic regression, classification trees, and LASSO regression produce more interpretable models but may underperform in predictive accuracy. Some authors propose human-centered modeling, which integrates explanatory data analysis, machine learning, and model interpretation within a feedback loop [17].

In this study, available tools were leveraged to interpret random forest results, thereby developing a clinically useful classification tree [18,19,20]. Additionally, effect sizes of relevant factors are reported to facilitate comparison with previous studies and to refine future research on known associations with CD.

The objectives of this study were twofold: (1) to conduct a scoping review analyzing published studies on cognitive decline (CD) screening in community pharmacies—focusing on screening methods and the role of pharmacists—in order to explore the available evidence on such programs and (2) to develop a patient profile to support early detection of cognitive decline.

## 2. Materials and Methods

### 2.1. Cognitive Impairment Screening in Community Pharmacy—Scoping Review

A scoping review of CD screening in community pharmacies was conducted according to Preferred Reporting Items for Systematic reviews and Meta-Analyses (PRISMA) guideline for scoping reviews [21].

#### 2.1.1. Literature Search

A search of publications between February 2015 and February 2025 was performed in three databases, including PubMed, Web of Science (WoS), and Scopus. The search strategy was as follows: (“Community Pharmacy Services” OR “Pharmacies”) AND (“Cognitive Dysfunction” OR “Mild Cognitive Impairment” OR “Subjective Cognitive Decline”).

The strategies used for each database are presented below:

PubMed: (“Community Pharmacy Services”[Mesh] OR “Pharmacies”[Mesh]) AND (“Cognitive Dysfunction”[Mesh] OR “Mild Cognitive Impairment”[Mesh] OR “Subjective Cognitive Decline”).

WoS: TS = (“Community Pharmacy” OR “Community Pharmacist” OR “Pharmacy Services” OR “Pharmacies”) AND TS = (“Cognitive Dysfunction” OR “Mild Cognitive Impairment” OR “Subjective Cognitive Decline” OR “Cognitive Decline” OR “Cognitive Impairment”)

Scopus: TITLE-ABS-KEY(“Community Pharmacy” OR “Community Pharmacist” OR “Pharmacy Services” OR “Pharmacies”) AND TITLE-ABS-KEY(“Cognitive Dysfunction” OR “Mild Cognitive Impairment” OR “Subjective Cognitive Decline” OR “Cognitive Decline” OR “Cognitive Impairment”).

#### 2.1.2. Eligibility Criteria

The inclusion criteria were as follows: cognitive screening interventions carried out in community pharmacies, studies that included screening results (e.g., number of patients assessed and percentage of positive screenings), and publications in English. The exclusion criteria included studies without involvement of community pharmacist, studies without screening results. Data were coded using a deductive and descriptive method.

#### 2.1.3. Data Extraction and Analysis

Study selection was initially conducted by one author, with a second author consulted in cases of uncertainty. Full-text screening was performed based on predefined inclusion and exclusion criteria. Disagreements were resolved through discussion between authors. A qualitative content analysis was conducted following the methodology outlined by Levac et al. [22], based on the Arksey and O’Malley framework. This approach includes six stages: identifying the research question, developing the search strategy, selecting studies, charting the data, collating and summarizing results, and consultation. These six steps guided the review process.

Variables included study type, country, population, number of patients, duration of the study, number of community pharmacies participating, screening method, and positive screening results.

### 2.2. Implementation of the Pharmaceutical Care Service for Subjective Cognitive Decline Screening

#### 2.2.1. Study Design

A cross-sectional study was conducted to evaluate the effectiveness of a CD screening program. Participants aged 50 years and older were eligible if they reported SMC, either directly by the patient or by a close relative or caregiver, and provided written informed consent. Individuals were excluded if they had a diagnosis of dementia, severe sensory impairments (e.g., blindness or deafness) that would preclude test administration, or physical disabilities that would interfere with testing procedures. A sample size of 233 participants was initially targeted to detect odds ratios of approximately 2.5 for relevant factors, assuming a significant level of 5% and 80% power [23]. Recruitment began in February 2022 and continued for 18 months. All participants provided written informed consent in accordance with the Declaration of Helsinki.

#### 2.2.2. Community Pharmacists’ Participation and Training

The Muy Ilustre Colegio Oficial de Farmacéuticos de Valencia (MICOF) invited pharmacies in Valencia to voluntarily participate. Pharmacists received training on dementia detection and the standardized work protocol.

#### 2.2.3. Subject Identification and Recruitment

Patients were recruited during routine pharmacy visits or through referrals from primary care physicians. The cognitive screening service was promoted via posters and informational brochures displayed in participating community pharmacies.

Pharmacists actively asked patients whether they experienced SMC, which could be reported either directly by the patient or by a close relative or caregiver. Only those who reported SMC were considered eligible for screening.

All participants provided informed consent prior to the assessment, which was conducted in a designated private area within the pharmacy to ensure confidentiality.

All selected subjects underwent cognitive decline screening, which was conducted by a trained pharmacist and consisted of administering three validated screening questionnaires. Patients with positive screening results—suggestive of potential cognitive decline—were referred to their primary care physician for further clinical evaluation.

#### 2.2.4. Assessment Tools for Cognitive Decline Screening (Table 1)

Three validated tests were used:

- Memory Impairment Screening (MIS) (max: 8; cutoff: ≤4; sensitivity: 74%; specificity: 96%) [24];
- Semantic Verbal Fluency (SVF) (cutoff: 10; sensitivity: 74%; specificity: 80%) [25,26];
- Short Portable Mental State Questionnaire (SPMSQ) (max: 10; cutoff: 3 errors; sensitivity: 85.7%; specificity: 79.3%) [27,28].

**Table 1:** General characteristics of the cognitive tests used. Adapted from Olazarán et al. 2016 [29].

Test	Duration (min)	Advantages	Disadvantages	Guidelines	Scope
MIS	4	Evaluates free and facilitated recall.	Requires the ability to read. Evaluates only memory.	CI detection	Primary Care
SVF	1	Friendly.	Does not evaluate EM. Requires a chronometer.	Complement of other tests	Primary Care Specialized Care
SPMSQ	3	Applicable to illiterate people.	Prior data must be known.	Dementia detection	Primary Care

MIS: Memory Impairment Screening; SVF: Semantic Verbal Fluency; SPMSQ: Short Portable Mental State Questionnaire; EM: Episodic Memory; CI: cognitive impairment.

A positive result in any test classifies patients as suspected CD cases. The cognitive screening tests were selected from among the various validated questionnaires currently available, based on their suitability for clinical application. This selection process was carried out with the guidance of the Valencian Society of Neurology [24] (Table 1).

The interview covered dementia risk factors according to the Lancet Commission (2020) [13] and the A-to-Z Dementia Knowledge List [30]. These variables included demographics (sex, family history of dementia, level of education), health conditions (hypertension, hypercholesterolemia, diabetes mellitus, depression and Herpes Virus Simplex (HVS) diagnosis and treatment, hearing loss and use of hearing aids, nutritional status, brain injury, benzodiazepine consumption, anticholinergic burden, Nonsteroidal Anti-Inflammatory Drugs (NSAIDs)), lifestyle habits variables (obesity, common tasks, living in a rural or urban area, playing a musical instrument, cognitive stimulation activities, internet and social networks use, reading, languages spoken, smoking, alcohol consumption, physical exercise, hours sleeping), and social and psychological variables (loneliness feelings, living alone, social contact).

Additional assessments:

- Fourteen-Point Mediterranean Diet Adherence Screen (MEDAS-14). Scale designed in the PREDIMED study [31,32];
- Five-Point Geriatric Depression Scale (GDS-5) (cutoff: ≥2; sensitivity: 82%; specificity: 98%) [33].

#### 2.2.5. Data Synthesis and Analysis

Data were collected using ATENFARMA^®^ version 5.3.3, a MICOF-developed platform.

The three cognitive tests were compared using McNemar’s test and Cohen’s Kappa to assess their contributions. Effect sizes of collected variables were calculated based on CD condition and *p*-values, using rank biserial correlation for quantitative variables. Depending on Shapiro–Wilk’s test, either an independent *t*-test or Mann–Whitney U test was applied. Qualitative variables were analyzed with chi-squared or Fisher’s exact test, with odds ratios as effect sizes. Finally, patient profiles for suspected CD were determined using the ranger package in R [34,35], employing a random forest with 50,000 trees. Variable importance was assessed through mean decreased accuracy and an improved Gini index [36].

## 3. Results

### 3.1. Cognitive Decline Screening in Pharmacies—Scoping Review

**Figure 1:**
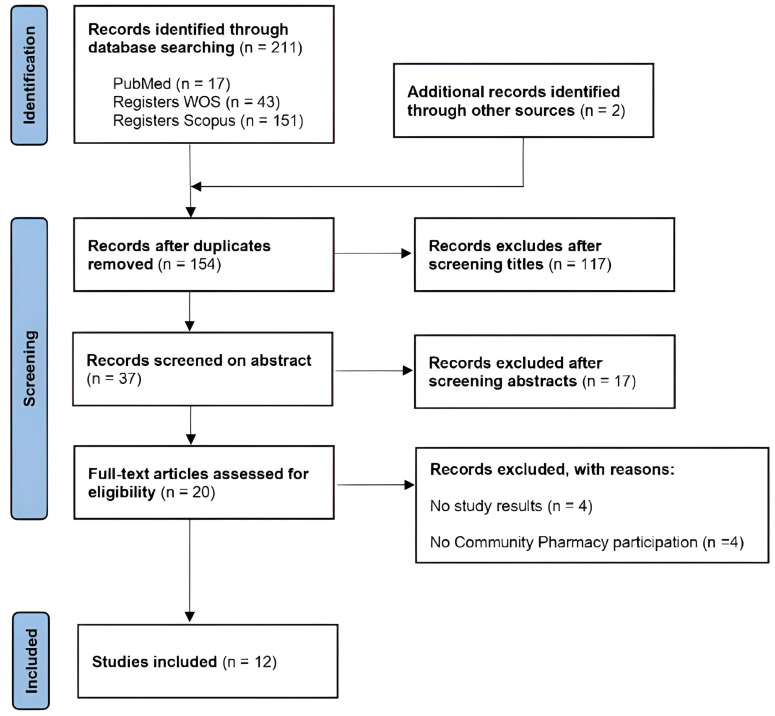
PRISMA flowchart of study selection process.

A total of 211 records were initially identified, and after screening and full-text evaluation, 12 studies met the inclusion criteria and were included in the review (Figure 1).

Table 2 presents the results of our data extraction, including first author, year of publication, type of study, country, type of population, number of patients screened, duration of the study, number of community pharmacies participating, screening method, and percentage of positive results after SCD screening.

The review identified studies conducted in Spain, Slovakia, and Italy [11,37,38,39,40,41,42,43,44,45,46,47]. Screening methodologies varied by country and target population.

### 3.2. Cognitive Decline Screening in Community Pharmacy as a Pharmaceutical Care Service

A total of 18 (1.45%) pharmacies adhered to our study, with their staff being trained to administer three cognitive tests: SVF, SPQMS, and MIS. After that training, they screened 286 patients with SMC who consented to participate in this study. In the collected sample, the average age is 71.1, a 74.8% are women, and the education level is divided into the following groups: only able to read and write (1%), primary (44.1%), secondary (19.9%), and higher education (25.9%). Among them, 79 (27.6%) were identified as suspected CD cases. With the data collected, a comparison of the cognitive test is presented, and a pairwise comparison of the tests is conducted using McNemar’s test (Table 3), revealing that the MIS/SPMSQ pair (*p*-value = 1) exhibited a similar error rate, with discordant classifications occurring in a comparable random manner. In contrast, the MIS/SVF (*p*-value = 0.028784) and SPMSQ/SVF (*p*-value = 0.014961) pairs demonstrated imbalanced discordant classifications, indicating that SVF has significantly different error rates than MIS and SPMSQ. Consequently, McNemar’s test suggests that SVF is classified differently from the other tests. However, Cohen’s kappa suggests that SVF exhibits greater agreement with MIS and SPMSQ than the agreement between MIS and SPMSQ themselves. Therefore, the three tests collect different groups of individuals with OCD, which indicates the complexity of the assessment landscape of the CD.

**Figure 2:**
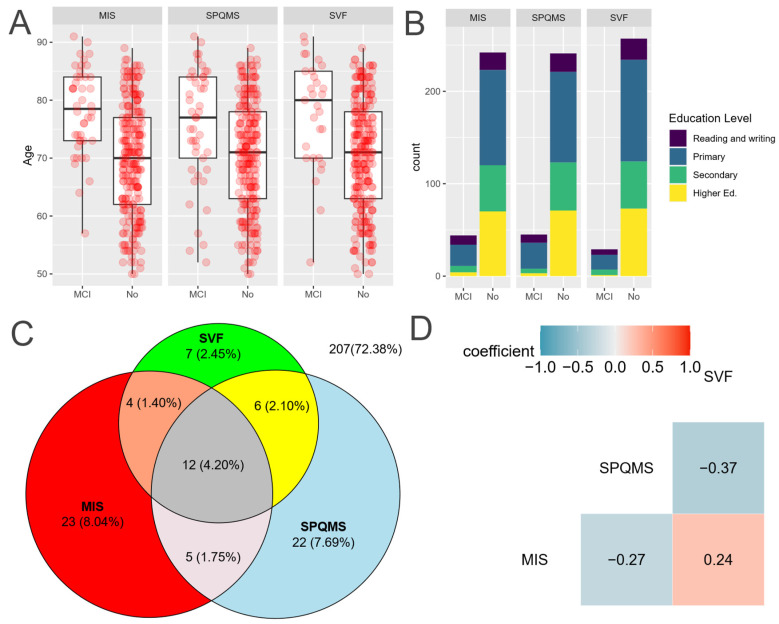
Comparison of the three cognitive tests. Source: Authors’ calculations with ATENFARMA database. (**A**) Boxplot of the score depending on the outcome for each test. (**B**) A stacked bar graph comparing the results for each test depending on the level of education. (**C**) A Venn diagram to visualize the level of agreement among the three tests. (**D**) Correlogram with Spearman’s correlation coefficient for the scores of the cognitive tests employed. MIS: Memory Impairment Screening; SVF: Semantic Verbal Fluency; SPMSQ: Short Portable Mental State Questionnaire; suspected MCI: mild cognitive impairment; Higher Ed: higher education.

As can be observed in Figure 2C, there are considerably fewer participants testing positive in SVF compared to the other tests. Box-and-whisker plots comparing age across the outcomes of various tests are illustrated in Figure 2A, while a stacked bar plot depicting educational levels is presented in Figure 2B. The agreement between the different cognitive tests is visualized with a Venn diagram in Figure 2C, revealing substantial differences in positive cases among the various tests. Additionally, a correlogram with Spearman’s correlation assesses the relationships between different scores.

### 3.3. Profile of the Patient Eligible for Cognitive Impairment Screening

In this section, the ATENFARMA database is analyzed to identify key characteristics of individuals with likely CD among SMC individuals by means of the importance indices produced by decision tree algorithms. To the completeness of the section and being able to compare with other studies, the estimation of the effect size is given.

Authors’ calculations with the ATENFARMA database. A: Odds ratio and its confidence interval at 95% confidence. The variables displayed are those whose false discovery rate is lower than 0.1 for an adequate test, and they are ordered according to odds ratio. B: Rank biserial correlation and its confidence interval at 95% confidence. The variables displayed are those whose false discovery rate is lower than 0.1 for an adequate test. C: Corrected Gini index for the variables whose false discovery rate is lower than 0.1.

**Figure 3:**
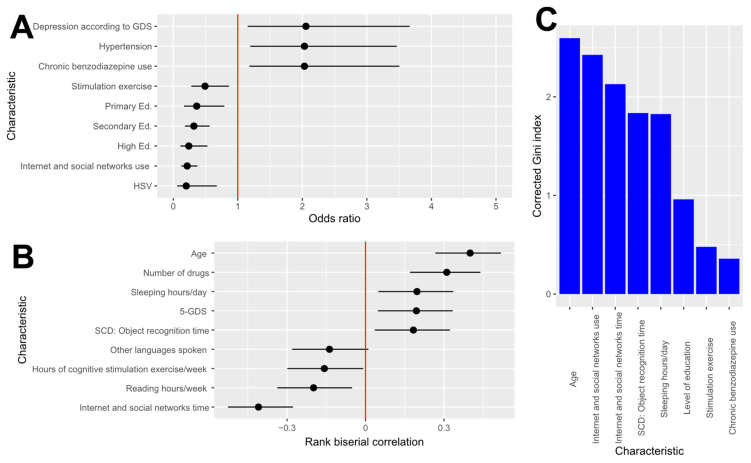
Statistical analysis of the characteristics in the ATENFARMA dataset. (**A**): Odd ratio and its confidence interval at 95% confidence. The variables displayed are those whose false discovery rate is lower than 0.1 for an adequate test and they are ordered according to odds ratio. (**B**): Rank biserial correlation and its confidence interval at 95% confidence. The variables displayed are those whose false discovery rate is lower than 0.1 for an adequate test (**C**): Corrected Gini index for the variables whose false discovery rate is lower than 0.1. GDS: Geriatric Depression Scale; Primary Ed: primary education; Secondary Ed: secondary education; High Ed: higher education; HSV: Herpes Simplex Virus; SCD: Subjective Cognitive Decline.

The qualitative variables collected in ATENFARMA whose false discovery rates are lower than 0.1 are presented in Table 4 (and Table A1 with all the variables), and the significant variables with a false discovery rate according to an appropriate test are presented in Figure 3A with the 95% confidence interval of the odds ratio (OR). As can be observed, the variables appearing are those associated with diseases and their treatments, such as depression (OR: 2.06), hypertension (OR: 2.03), or the use of benzodiazepines (OR: 2.03).

On the other hand, variables with a low odds ratio include herpes virus (OR: 0.20), family history of dementia (OR: 0.51), and certain cognitive skill-related factors such as education level (Primary ed. OR: 0.36; Secondary Ed. OR:0.32; Higher Ed. OR: 0.24), use of social networks (OR: 0.22), or engagement in stimulation exercises (OR: 0.49). Remarkably, all the participants treated for herpes had normal cognition. For more information, a comprehensive table with all variables is provided in Table A2.

The effect size of the quantitative variables was assessed using the rank biserial correlation (r), given the inability to assume normality for most variables and the consequent use of non-parametric tests for most of them. In Figure 3B, a graphical summary of the most significant variables is displayed, and Table 5 contains information on the variable whose false discovery rate is lower than 0.1, and all the aggregate values are provided in Table A2.

Among the variables exhibiting a negative correlation with CD are those related to the duration of cognitive activities, such as internet use (r: −0.41), reading (r: −0.20), or engagement in stimulation exercises (r: −0.16). Conversely, factors exhibiting a positive correlation with CD primarily include age (r: 0.40), the number of chronic medications (r: 0.31), total hours of sleep (r: 0.20), the depression test GDS-5 (r: 0.19), and the duration of problems with object recognition (r: 0.18). In Table A3, all the numerical information is provided.

The main tool proposed to determine the relevance of variables in identifying individuals for screening is decision trees. For that, a random forest consisting of 500,000 decision trees was trained, and after that, the importance of the variables by means of an unbiased impurity Gini’s index (MDcI) and the mean decrease in accuracy (MDA) were calculated. As a result of that analysis, variables with a significantly large unbiased impurity index after running the random forest are detailed in Figure 2C and Table 6. According to the information in that graphic, age (MDcI: 2.60) emerges as the variable with the highest discrimination of cases, followed by social network usage (MDcI: 2.43); sleep duration (MDcI: 1.83); cognitive activity-related variables, such as attained education level (MDcI: 0.96); and the presence of diseases such as depression (MDcI: 0.54) or adherence to chronic treatments such as benzodiazepine (MDcI: 0.36).

Notably, Body Mass Index (BMI) exhibits a substantial change between the unbiased impurity index and the conventional one. While BMI ranks as the second variable with the highest impurity index (7.66), the correction index results in a reduced impurity index (MDcI: 0.32). For completeness, Table A4 and Table A5 are tables containing the unbiased impurity index and the mean decrease in accuracy.

**Figure 4:**
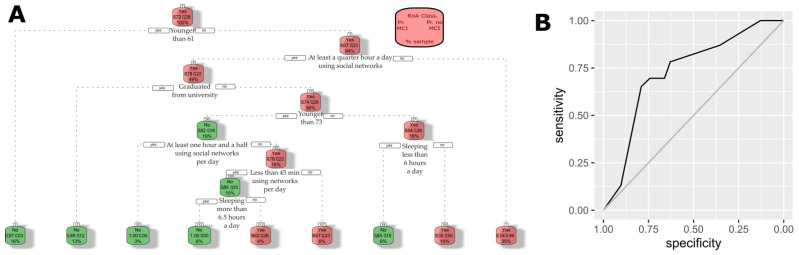
Selected decision tree. Authors’ calculations with the ATENFARMA database. (**A**) Decision tree selected. (**B**) ROC curve of the selected test assessed with 30% of the ATENFARMA sample.

A set of 50 decision trees was trained with 70% of the dataset and evaluated with the remaining 30% of the dataset. As a summary of all these trees, the average AUC is 0.6533, the average sensitivity is 0.7086, the average specificity is 0.5269, and the average of predicted cases is 53.63%. Thoroughly, the authors considered the most convenient to be implemented in community pharmacies, which is showcased in Figure 4. In summary, the decision tree recommends focusing on individuals aged 61 or older who do not engage in social networks. For those who are active on social networks, the screening should also encompass individuals over 73 years of age, with lower educational attainment, and a sleep duration exceeding 6 h. With respect to the performance of that tree, the estimated AUC is 0.7297, and a 95% confidence interval of the AUC for using Delong’s method is (0.6129, 0.8464). For the other measures of performance, its estimated sensitivity is 0.6957, its estimated specificity is 0.6613, and the percentage of predicted cases is 43.53%. In conclusion, applying such a tree reduced the number of individuals to take screen tests while keeping most of the positive cases.

## 4. Discussion

SMC is a good predictor of dementia as it is one of the first alert signs [4,48]. It has been shown that there is an association between SMC and an increase in the 3-year incidence of cognitive impairment [49] and a decline in global cognition over 6 years [50]. Previous studies have regarded SMC as an important indicator of testing positive in CD screening [11,40], and our results align with this.

Primary care provides a longitudinal perspective of patients, crucial for early MCI detection. As such, it is the ideal setting for detecting and initiating the first management of SCD [29]. Specifically, community pharmacists have shown a high level of interest and relevance in the field, but there is also a noticeable knowledge gap that prevents them from feeling comfortable when intervening with patients with or at risk of dementia [16]. In a previous study of our group, we found that younger pharmacists had better knowledge of risk factors associated with dementia. The risk factors most often identified were a family history of dementia, followed by social isolation, and the least known protective factors were internet use, avoidance of pollution, and the use of anti-inflammatory drugs [30].

While studies on the detection of suspected MCI in community pharmacy are limited, the significance of CD screening using psychometric tests in primary care has been emphasized. Additionally, the promotion of cognitive training programs to develop preventive strategies and compensatory measures for mitigating cognitive impairment in old age has been highlighted [15,21].

Despite certain obstacles surrounding Pharmaceutical Care Services, such as a lack of time, shortage of staff, or difficulties with reimbursement, the interest in these roles is increasing [51,52]. An example of this is smoking cessation provided in community pharmacies [53]. Moreover, community pharmacists are among the most accessible healthcare professionals regularly visited in primary care settings. Therefore, they can play a vital role in the early detection of MCI. In this regard, screening can be easily implemented into the Pharmaceutical Care Services offered during the daily practice [14]. In addition, the development and improved accessibility of valuable tools and training would better equip community pharmacists in the management of patients [16].

The scoping review highlights the heterogeneity of MCI screening approaches in community pharmacies, complicating study comparability and hindering the development of standardized recommendations. The diversity in screening methods and target populations underscores the need for unified protocols to optimize patient detection and referral. Community pharmacy cognitive screening exhibits strong external validity, demonstrating its potential for widespread application across diverse populations and settings [11,37,38,39,40,41,42,43,44,45,46,47]. This supports the feasibility of implementing standardized protocols across diverse contexts. Nevertheless, while screening in community pharmacies is feasible, effective implementation requires specialized pharmacist training, digital tools for data recording, and enhanced collaboration with primary care.

In this context, our study aimed to develop a detailed profile of patients who are candidates for CD screening in community pharmacies, directly addressing the lack of studies providing clear criteria on which patient characteristics most likely necessitate screening. By developing this profile, we aim to facilitate early intervention implementation and enhance the understanding of pharmacists’ roles in dementia detection.

Our results suggest that, in addition to age, limited use of the internet or social media, a suboptimal sleep pattern (less than 5 or more than 10 h per day), and a lower educational level are key factors that may indicate greater vulnerability to cognitive decline. These findings indicate that individuals with these characteristics are ideal candidates for cognitive decline screening in community pharmacies, which could support early detection of dementia and improve prevention and intervention strategies.

As it is well known, the age and educational level of the participants are among well-established factors showing a stronger association with testing positive on cognitive assessments, which convert them into potential confounding variables for additional associations [54]. Likewise, a high level of education, reading, and daily internet use are factors that have been associated with a reduced risk of receiving positive scores compatible with suspected CD [39]. Less time in education is defined by Livingston and colleagues as no secondary school education. The low level of education holds the second Population-Attributable Fraction in the Lancet Commission model [13]. It is believed to contribute to a lower cognitive reserve, although it has not yet been clearly defined whether education beyond secondary level provides additional protection. It has also been reported that there is an association between sleep duration and the risk of CD or dementia, with a higher risk of dementia observed in individuals who sleep more than 10 h or less than 5 h [13].

Our decision tree analysis indicates that CD screening should be strategically targeted to distinct risk profiles. Specifically, individuals aged 61 and older who do not engage in internet or social media activities represent a high-risk group, while among those who are active online, the risk extends to individuals over 73, particularly when combined with lower educational attainment and sleep durations exceeding 6 h. These insights, which corroborate the findings of Ramos et al. [39], emphasize the importance of tailoring screening protocols to modifiable lifestyle and demographic factors. Such an approach can enhance early detection efforts, particularly in community pharmacy settings where accessible, targeted interventions can significantly impact public health outcomes.

Early intervention, involving pharmacological treatments and cognitive stimulation, may slow disease progression and improve patient prognosis and quality of life. Consequently, diagnosing the disease in its early stages is crucial [30]. In this study, assessments were designed to be as specific and personalized as possible, requiring approximately 30 min and providing comprehensive information on patients’ cognitive status and associated risk factors. The number of patients identified with suspected CD was comparable across screening tools. However, incorporating multiple questionnaires expanded detection capabilities and improved the overall accuracy of the screening process.

## 5. Strengths and Limitations

Despite the promising findings, this study has several limitations. Regarding the scoping review, the analysis identified limited research on the early detection of dementia through CD screening in community pharmacies. The efficiency of these screening initiatives may be compromised when pharmacists perform them alongside their routine tasks, in contrast to dedicated screening programs described in previous studies. Overall, pharmacy participation was limited, suggesting barriers to the implementation of these programs. This gap in scientific evidence highlights the need for further research in this area.

As for the development of the patient profile, this study was conducted within the context of community pharmacies—a setting with high accessibility and frequent patient interaction. The profile provides actionable insights that may help pharmacists identify individuals at risk of cognitive impairment, contributing to public health efforts in dementia prevention and intervention. The inclusion of SMC as a variable reflects the current understanding that it may serve as an early marker of cognitive decline, especially in the absence of objective deficits, and adds value to early detection strategies.

However, the cross-sectional nature of the empirical component limits the ability to establish causality or assess progression from SMC to objective cognitive decline. Additionally, reliance on self-reported memory complaints may introduce reporting bias, particularly among older adults who may underreport or misinterpret their symptoms. Without longitudinal follow-up or clinical confirmation, the predictive value of the proposed patient profile remains hypothetical and requires validation in larger or longitudinal studies.

## 6. Conclusions

CD detection in community pharmacies is a promising strategy but faces challenges regarding standardization and applicability. Future research should focus on assessing the clinical impact of screening, patient acceptance, and its integration into routine pharmaceutical practice. Targeting individuals with limited digital engagement, poor sleep, and lower education could enhance early dementia detection.

## Figures and Tables

**Table 2:** Characteristics of the studies included.

Citation	Study Type	Country	Population	n	Duration	Number of Community Pharmacies Participating	Screening Method	Positive Screening Results
Climent, 2015 [37]	Cross-sectional observational	Spain	Community population aged ≥65	729	3 months	14	SPMSQ, MMSE	17.6%
Climent, 2018 [11]	Cross-sectional	Spain	Non-institutionalized people aged ≥65 who went regularly to the pharmacy	728	1 year	14	SPMSQ, MMSE	17.4%
Feijoo, 2019 [38]	Observational transversal	Spain	Non-institutionalized patients aged ≥65 in the community pharmacy setting	729	2 years	13	SPMSQ, MMSE	17.6%
Ramos, 2021 [39]	Comparative	Spain	Community population aged ≥50 with SMC	281	1 year	19	MIS, SPMSQ, SVF	29.84%
Ramos, 2021 [40]	Cross-sectional	Spain	Adults aged >50 years with SMC	497	17 months	19	MIS, SPMSQ, SVF	30.8%
Mačeková, 2022 [41]	Pilot	Slovakia	Patients aged ≥60 who visited the community pharmacies	222	1 year	16	MoCA	41%
Ortiz, 2023 [42]	Cross-sectional observational	Spain	Caregivers of persons aged >70 not previously diagnosed with CI and not living in a nursing home or hospitalized	910		197	IQ-CODE	38.5%
Bragazzi, 2023 [43]	Observational, cross-sectional, multicenter	Italy	Clients attending a network of community pharmacies that had agreed to offer neuropsychological screening	185			MoCA, BSRT, ROCF	48.6%, 10.3–8.6%, 21.1%
Macekova 2023 [44]	Cross-sectional observational	Slovakia	People who visited the community pharmacies	222	1 year	16	MoCA	(18.4 ± 6.0) (23.6 ± 4.3)
Martínez 2023 [45]	Observational descriptive cross-sectional	Spain	People who attended or requested the services of the community pharmacy aged >50 years old and reporting SMC, either explicitly expressed by the patient or identified through indirect questions from the pharmacist	39	2 months	1	MIS, SVF, SPMSQ	28.2%
Putignano, 2024 [46]	Cross-sectional-assisted	Italy	Subjects aged >60 years old who regularly go to local pharmacies	279		17	MMSE	17.9%
García-Lluch, 2024 [47]	Cross-sectional	Spain	Community population aged ≥50 with SMC	172	4 years		MIS, SVF, SPMSQ	60%

MMSE: Mini-Mental State Examination; SPMSQ: Short Portable Mental State Questionnaire; MIS: Memory Impairment Screen; SVF: Semantic Verbal Fluency; IQ-CODE: Informant Questionnaire on Cognitive Decline; SMC: Subjective Memory Complaint; MoCA: Montreal Cognitive Assessment; BSRT: Babcock Story Recall Test; ROCF: Rey–Osterrieth complex figure; sMetS: Suspected Metabolic Syndrome.

**Table 3:** Pairwise comparison of the three cognitive tests using McNemar’s test and Cohen’s Kappa.

Tests	Statistic ^1^	*p*-Value ^2^	Kappa ^3^	CI ^4^
MIS/SPQMS	0.00	1.000	0.27	[0.12, 0.41]
SPQMS/SVF	5.92	0.015	0.41	[0.26, 0.57]
MIS/SVF	4.78	0.030	0.361	[0.21, 0.51]

Source: Author’s calculation using ATENFARMA database. ^1^ Statistic of McNemar’s test; ^2^ *p*-value of McNemar’s test. ^3^ Point estimation of Cohen’s Kappa. ^4^ Point estimation of Cohen’s Kappa. MIS: Memory Impairment Screening; SVF: Semantic Verbal Fluency; SPMSQ: Short Portable Mental State Questionnaire; CI: cognitive impairment.

**Table 4:** Odds ratio of the variables collected in ATENFARMA with a significance level (α = 0.1) and the variable HSV treatment.

Characteristic	Odds Ratio (OR) (95% CI)	n	*p*-Value (Test)	FDR
Depression according to GDS	2.06 (1.15, 3.66)	286	0.020 * (**χ**^2^)	0.08514
Hypertension	2.03 (1.19, 3.46)	286	0.012 * (**χ**^2^)	0.08514
Chronic benzodiazepine use	2.03 (1.18, 3.50)	286	0.015 * (**χ**^2^)	0.08514
Stimulation exercise	0.49 (0.28, 0.86)	286	0.018 * (**χ**^2^)	0.08514
Primary Ed.	0.36 (0.17, 0.79)	286	0.016 * (**χ**^2^)	0.08514
Secondary Ed.	0.32 (0.18, 0.56)	286	9.703 × 10^−5^ *** (**χ**^2^)	0.001892
High Ed.	0.24 (0.11, 0.53)	286	0.000 *** (**χ**^2^)	0.004047
Internet and social networks use	0.22 (0.12, 0.38)	286	3.843 × 10^−8^ *** (**χ**^2^)	1.499 × 10^−6^
HSV	0.20 (0.06, 0.67)	286	0.003 ** (F)	0.02901
HSV treatment	0.00 (-)	37	0.105 (F)	0.2275

* *p*-value < 0.05; ** *p*-value < 0.01; *** *p*-value < 0.001. GDS: Geriatric Depression Scale; Primary Ed: primary education; Secondary Ed: secondary education; High Ed: higher education; HSV: Herpes Virus Simplex.

**Table 5:** Effect sizes of the quantitative variables collected in ATENFARMA whose false discovery rate is lower than 0.1 (significance level α = 0.1).

Characteristic	Rank Biserial Correlation	CD		No CD		*p*-Value
		n	$\bar{x}\pm s$	n	$\bar{x}\pm s$	
Internet and social networks time	−0.41 (−0.53, −0.28)	79	0.51 ± 0.82	207	1.42 ± 1.69	3.906 × 10^−8^ *** (MW)
Reading hours/week	−0.20 (−0.34, −0.05)	79	2.63 ± 4.16	207	5.36 ± 8.13	0.008 ** (MW)
Hours of cognitive stimulation exercise/week	−0.16 (−0.30, −0.01)	79	3.41 ± 7.02	207	4.88 ± 6.96	0.019 * (MW)
Other languages spoken	−0.14 (−0.28, 0.01)	79	0.42 ± 0.50	207	0.56 ± 0.50	0.037 * (MW)
SCD: Time	0.06 (−0.09, 0.21)	79	16.70 ± 17.12	207	18.63 ± 23.94	0.443 (MW)
SCD: Language alteration time	0.11 (−0.04, 0.25)	79	4.63 ± 12.62	207	4.29 ± 15.52	0.053 (MW)
SCD: Object recognition time	0.18 (0.04, 0.32)	79	7.76 ± 17.38	207	2.60 ± 12.20	0.000 *** (MW)
5-GDS	0.19 (0.05, 0.33)	79	1.43 ± 1.36	207	1.00 ± 1.17	0.007 ** (MW)
Sleeping hours/day	0.20 (0.05, 0.34)	79	7.46 ± 2.40	207	6.73 ± 1.55	0.00894 ** (MW)
Number of drugs	0.31 (0.17, 0.44)	79	5.63 ± 3.36	207	4.02 ± 3.32	4.421 × 10^−5^ *** (MW)
Age	0.40 (0.27, 0.52)	79	75.96 ± 8.71	207	69.30 ± 9.44	1.712 × 10^−7^ *** (MW)

The columns are the rank biserial correlation of the characteristic with a 95% CI, the number of individuals with CD, the mean and standard deviation of the group with DC, the number of individuals without CD, the mean and standard deviation of the group without CD, and the *p*-value of the test indicated between brackets joint to the significance levels. The performed tests are Mann–Whitney’s U test (MW) and *t*-test of two independent samples assuming non-identical variances (*t*-test). For the significance level, the following symbols are used: *p*-value < 0.01; * *p*-value < 0.05; ** *p*-value < 0.01; *** *p*-value < 0.001. SCD: Subjective Memory Complaint; 5-GDS: 5-Point Geriatric Depression Scale.

**Table 6:** Results of the tests of importance based on the corrected Gini index of the variables collected in ATENFARMA for the variables with lower FDR.

Characteristics	MDcI	MDI	*p*-Value	FDR
Age	2.60	9.70	0.010 **	0.1089
Internet and social networks use	2.42	3.78	0.010 **	0.1089
Sleeping hours/day	1.83	6.818	0.010 **	0.1089
Chronic benzodiazepine use	0.36	1.35	0.020 *	0.1089
Stimulation exercise	0.48	1.95	0.020 *	0.1089
SCD: Object recognition time	1.84	4.05	0.020 *	0.1089
Level of education	0.96	3.26	0.020 *	0.1089
Internet and social networks time	2.13	5.40	0.020 *	0.1089

The columns are the name of the corresponding characteristics followed by four measures of importance: the corrected mean decrease in the corrected impurity (MDcI), the mean decrease in the original Gini’s impurity decreased (MDI), the *p*-value by means of the Altmann’s method, and the false discovery rate (FDR). For the significance level, the following symbols are used: * *p*-value < 0.05; ** *p*-value < 0.01; SCD: Subjective Cognitive Decline.

## Data Availability

The datasets generated during and/or analyzed during the current study are available from the corresponding author on reasonable request.

## Abbreviations

The following abbreviations are used in this manuscript:

BMI	Body Mass Index
CD	Cognitive Decline
GDS-5	5-Point Geriatric Depression Scale
HVS	Herpes Virus Simplex
MCI	Mild Cognitive Impairment
MEDAS-14	14-Point Mediterranean Diet Adherence Screen
MICOF	in Spanish Muy Ilustre Colegio Oficial de Farmacéuticos de Valencia
MIS	Memory Impairment Screening
NSAIDs	Nonsteroidal Anti-Inflammatory Drugs
OCD	Objective Cognitive Decline
PRISMA	Preferred Reporting Items for Systematic reviews and Meta-Analyses
SCD	Subjective Cognitive Decline
SPMSQ	Short Portable Mental State Questionnaire
SVF	Semantic Verbal Fluency
SMC	Subjective Memory Complaint
WoS	Web of Science

## Appendix A

**Table A1:** Odds ratio and relative risk of the variables collected in ATENFARMA.

Characteristic	Odds Ratio OR (95% CI)	Relative Risk RR (95% CI)	n	Exposed Odds (%)	Unexposed Odds (%)	*p*-Value (Test)	FDR
Hypertension treatment	-	-	145	50/94 (34.72)	0/1 (0.00)	1 (F)	1
Hypercholesterolemia treatment	4.30 (0.94, 19.62)	3.10 (0.83, 11.61)	146	47/82 (36.43)	2/15 (11.76)	0.055 (F)	0.1343
N05CD ATC Code CD	3.26 (1.06, 10.01)	2.04 (1.19, 3.51)	286	7/6 (53.85)	72/201 (26.37)	0.051 (F)	0.1323
Malnutrition	2.83 (0.66, 12.04)	1.91 (0.89, 4.09)	119	4/4 (50.00)	29/82 (26.13)	0.215 (F)	0.3731
Brain injury	2.42 (0.85, 6.91)	1.76 (0.99, 3.12)	286	7/8 (46.67)	72/199 (26.57)	0.133 (F)	0.2507
Loneliness feeling	2.13 (0.78, 5.85)	1.57 (0.88, 2.79)	71	12/12 (50.00)	15/32 (31.91)	0.220 (**χ**^2^)	0.3731
Diabetes mellitus	2.08 (1.10, 3.95)	1.64 (1.10, 2.45)	286	20/29 (40.82)	59/178 (24.89)	0.036 * (**χ**^2^)	0.1011
Depression according to GDS	2.06 (1.15, 3.66)	1.71 (1.10, 2.67)	286	59/122 (32.60)	20/85 (19.05)	0.020 * (**χ**^2^)	0.08514
Hypertension	2.03 (1.19, 3.46)	1.68 (1.13, 2.49)	286	50/95 (34.48)	29/112 (20.57)	0.012 * (**χ**^2^)	0.08514
Chronic benzodiazepine use	2.03 (1.18, 3.50)	1.64 (1.13, 2.37)	286	33/54 (37.93)	46/153 (23.12)	0.015 * (**χ**^2^)	0.08514
N05BA ATC Code	1.92 (1.10, 3.36)	1.57 (1.08, 2.29)	286	29/48 (37.66)	50/159 (23.92)	0.031 * (**χ**^2^)	0.1011
Alone	1.92 (1.09, 3.41)	1.57 (1.08, 2.30)	286	27/44 (38.03)	52/163 (24.19)	0.035 * (**χ**^2^)	0.1011
Hypercholesterolemia	1.85 (1.09, 3.15)	1.57 (1.06, 2.32)	286	49/97 (33.56)	30/110 (21.43)	0.030 * (**χ**^2^)	0.1011
Adherence MEDAS	1.44 (0.72, 2.86)	1.29 (0.81, 2.04)	286	15/29 (34.09)	64/178 (26.45)	0.390 (**χ**^2^)	0.6081
Depression	1.27 (0.70, 2.30)	1.18 (0.78, 1.80)	286	21/46 (31.34)	58/161 (26.48)	0.534 (**χ**^2^)	0.8006
Depression treatment	1.23 (0.66, 2.31)	1.16 (0.75, 1.80)	286	18/40 (31.03)	61/167 (26.75)	0.627 (**χ**^2^)	0.8728
Obesity	1.18 (0.60, 2.31)	1.13 (0.69, 1.83)	179	20/48 (29.41)	29/82 (26.13)	0.760 (**χ**^2^)	0.9833
Diabetes mellitus control	1.18 (0.25, 5.62)	1.11 (0.42, 2.90)	49	17/24 (41.46)	3/5 (37.50)	1 (F)	1
Rural vs. urban	1.14 (0.63, 2.06)	1.10 (0.73, 1.64)	225	26/54 (32.50)	43/102 (29.66)	0.770 (**χ**^2^)	0.9833
Universal task	1.14 (0.57, 2.26)	1.10 (0.67, 1.78)	286	14/33 (29.79)	65/174 (27.20)	0.854 (**χ**^2^)	1
BMI	1.01 (0.54, 1.86)	1.01 (0.64, 1.58)	210	26/73 (26.26)	29/82 (26.13)	1 (**χ**^2^)	1
Overweight	1.01 (0.54, 1.86)	1.01 (0.64, 1.58)	210	26/73 (26.26)	29/82 (26.13)	1 (**χ**^2^)	1
Sex	0.99 (0.55, 1.80)	0.99 (0.64, 1.53)	286	59/155 (27.57)	20/52 (27.78)	1 (**χ**^2^)	1
Hearing aid use	0.92 (0.27, 3.12)	0.94 (0.38, 2.34)	110	4/12 (25.00)	25/69 (26.60)	1 (F)	1
Hearing loss	0.90 (0.53, 1.54)	0.93 (0.63, 1.37)	286	29/81 (26.36)	50/126 (28.41)	0.810 (**χ**^2^)	0.9872
Hypercholesterolemia control	0.82 (0.28, 2.42)	0.88 (0.45, 1.74)	146	43/87 (33.08)	6/10 (37.50)	0.782 (F)	0.9833
Playing a musical instrument	0.78 (0.21, 2.90)	0.83 (0.30, 2.28)	286	3/10 (23.08)	76/197 (27.84)	1 (F)	1
Anticholinergic drugs	0.68 (0.22, 2.12)	0.75 (0.31, 1.83)	286	4/15 (21.05)	75/192 (28.09)	0.605 (F)	0.8728
Family history of dementia	0.51 (0.29, 0.90)	0.61 (0.40, 0.94)	286	22/89 (19.82)	57/118 (32.57)	0.027 * (**χ**^2^)	0.1011
NSAIDs	0.51 (0.22, 1.21)	0.60 (0.30, 1.20)	286	7/33 (17.50)	72/174 (29.27)	0.132 (F)	0.2507
Stimulation exercise	0.49 (0.28, 0.86)	0.59 (0.38, 0.91)	286	22/91 (19.47)	57/116 (32.95)	0.018 * (**χ**^2^)	0.08514
Social contact	0.42 (0.15, 1.13)	0.58 (0.30, 1.10)	71	9/24 (27.27)	18/20 (47.37)	0.135 (**χ**^2^)	0.2507
Primary Ed.	0.36 (0.17, 0.79)	0.52 (0.34, 0.81)	286	65/192 (25.29)	14/15 (48.28)	0.016 * (**χ**^2^)	0.08514
Secondary Ed.	0.32 (0.18, 0.56)	0.43 (0.28, 0.67)	286	21/110 (16.03)	58/97 (37.42)	9.703 × 10^−5^ *** (**χ**^2^)	0.001892
Hypertension control	0.26 (0.02, 2.89)	0.51 (0.22, 1.17)	145	48/94 (33.80)	2/1 (66.67)	0.273 (F)	0.444
High Ed.	0.24 (0.11, 0.53)	0.32 (0.16, 0.64)	286	8/66 (10.81)	71/141 (33.49)	0.000 *** (**χ**^2^)	0.004047
Internet and social networks use	0.22 (0.12, 0.38)	0.35 (0.24, 0.51)	286	34/161 (17.44)	45/46 (49.45)	3.843 × 10^−8^ *** (**χ**^2^)	1.499 × 10^−6^
HSV	0.20 (0.06, 0.67)	0.27 (0.09, 0.80)	286	3/34 (8.11)	76/173 (30.52)	0.003 ** (F)	0.02901
HSV treatment	0.00 (-)	0.00 (-)	37	0/19 (0.00)	3/15 (16.67)	0.105 (F)	0.2275

The name of the characteristic, the odds ratio and its confidence interval at 95%, the relative risk and its confidence interval, odds of CD as a fraction and percentage of CD for individuals exposed to the characteristic, odds of CD as a fraction and percentage of CD for individuals unexposed to the characteristic, *p*-value, and the applied test and FDR. The performed tests are chi-squared (**χ**^2^) and Fisher’s exact test (F). For the significance level (α), the following symbols are used: *p*-value < 0.01, * *p*-value < 0.05, ** *p*-value < 0.01, and *** *p*-value < 0.001. Please note that the study population is individuals older than 50 with Subjective Cognitive Decline. GDS: Geriatric Depression Scale; ATC: Anatomical Therapeutic Chemical classification system; MEDAS: Mediterranean Diet Adherence Screen; BMI: Body Mass Index; NSAIDs: Nonsteroidal Anti-Inflammatory Drugs; Primary Ed: Primary Education, Secondary Ed: Secondary Education; High Ed: higher education; HSV: Herpes Simplex Virus; CD: Cognitive Decline.

**Table A2:** Effect sizes of the quantitative variables collected in ATENFARMA.

Characteristic	Rank Biserial Correlation	CD		No CD		*p*-Value
		n	$\bar{x}\pm s$	n	$\bar{x}\pm s$	
Internet and social networks time	−0.41 (−0.53, −0.28)	79	0.51 ± 0.82	207	1.42 ± 1.69	3.906 × 10^−8^ *** (MW)
Reading hours/week	−0.20 (−0.34, −0.05)	79	2.63 ± 4.16	207	5.36 ± 8.13	0.008 ** (MW)
Hours of cognitive stimulation exercise/week	−0.16 (−0.30, −0.01)	79	3.41 ± 7.02	207	4.88 ± 6.96	0.019 * (MW)
Other languages spoken	−0.14 (−0.28, 0.01)	79	0.42 ± 0.50	207	0.56 ± 0.50	0.037 * (MW)
Number of alcohol units/week	−0.13 (−0.27, 0.02)	79	1.80 ± 3.18	207	2.20 ± 3.21	0.075 (MW)
MEDAS-14	−0.09 (−0.24, 0.06)	79	8.19 ± 2.49	207	8.56 ± 2.14	0.234 (MW)
Number of cigarettes/day	−0.08 (−0.23, 0.07)	79	0.92 ± 3.74	207	1.81 ± 5.00	0.070 (MW)
Hours of physical exercise/week	−0.06 (−0.20, 0.09)	79	4.62 ± 6.47	207	5.36 ± 6.89	0.444 (MW)
Number of years without smoking	−0.02 (−0.31, 0.28)	18	20.67 ± 13.35	65	21.03 ± 13.61	0.920 (*t*-test)
BMI	−0.02 (−0.17, 0.13)	79	26.71 ± 5.36	207	26.90 ± 4.89	0.784 (*t*-test)
Overweight	−0.02 (−0.17, 0.13)	79	26.71 ± 5.36	207	26.90 ± 4.89	0.784 (*t*-test)
Hours of playing a musical instrument/week	−0.02 (−0.17, 0.13)	79	0.35 ± 2.48	207	0.37 ± 1.85	0.488 (MW)
Hearing loss evolution (months)	−0.01 (−0.25, 0.23)	29	23.14 ± 17.07	81	22.65 ± 16.77	0.932 (MW)
Number of friends seen during the last week	0.03 (−0.25, 0.30)	27	2.30 ± 3.23	44	1.95 ± 2.85	0.844 (MW)
SCD: Time	0.06 (−0.09, 0.21)	79	16.70 ± 17.12	207	18.63 ± 23.94	0.443 (MW)
SCD: Language alteration time	0.11 (−0.04, 0.25)	79	4.63 ± 12.62	207	4.29 ± 15.52	0.053 (MW)
SCD: Object recognition time	0.18 (0.04, 0.32)	79	7.76 ± 17.38	207	2.60 ± 12.20	0.000 *** (MW)
5-GDS	0.19 (0.05, 0.33)	79	1.43 ± 1.36	207	1.00 ± 1.17	0.007 ** (MW)
Sleeping hours/day	0.20 (0.05, 0.34)	79	7.46 ± 2.40	207	6.73 ± 1.55	0.009 ** (MW)
Number of drugs	0.31 (0.17, 0.44)	79	5.63 ± 3.36	207	4.02 ± 3.32	4.421 × 10^−5^ *** (MW)
Age	0.40 (0.27, 0.52)	79	75.96 ± 8.71	207	69.30 ± 9.44	1.712 × 10^−7^ *** (MW)

The columns are the rank biserial correlation of the characteristic with a 95% CI, the number of individuals with CD, the mean and standard deviation of the group with DC, the number of individuals without CD, the mean and standard deviation of the group without CD, and the *p*-value of the test indicated between brackets joint to the significance levels. The performed tests are Mann–Whitney’s U test (MW) and *t*-test of two independent samples assuming non-identical variances (*t*-test). For the significance level, the following symbols are used: *p*-value < 0.01, * *p*-value < 0.05, ** *p*-value < 0.01, and *** *p*-value < 0.001. Please note that the study population is individuals older than 50 with Subjective Cognitive Decline. MEDAS-14: 14-Point Mediterranean Diet Adherence Screen; BMI: Body Mass Index; SCD: Subjective Cognitive Decline; 5-GDS: 5-Point Geriatric Depression Scale.

**Table A3:** Results of the tests of importance based on the corrected Gini index of the variables collected in ATENFARMA.

Characteristics	MDcI	MDI	*p*-Value	FDR
Age	2.595	9.696	0.010 **	0.1089
Internet and social networks use	2.425	3.779	0.010 **	0.1089
Sleeping hours/day	1.825	6.812	0.010 **	0.1089
Chronic benzodiazepine use	0.3589	1.349	0.020 *	0.1089
Stimulation exercise	0.4791	1.953	0.020 *	0.1089
SCD: Object recognition time	1.835	4.048	0.020 *	0.1089
Level of education	0.9602	3.256	0.020 *	0.1089
Internet and social networks time	2.128	5.399	0.020 *	0.1089
5-GDS	0.5481	3.064	0.03 *	0.1452
Number of drugs	0.9068	5.316	0.050 *	0.1815
Hypertension	0.3087	1.553	0.050 *	0.1815
N05BA ATC Code	0.2671	1.32	0.050 *	0.1815
Other languages spoken	0.2014	1.404	0.059	0.1906
Hypercholesterolemia treatment	0.276	1.682	0.069	0.1906
Family history of dementia	0.2439	1.692	0.069	0.1906
N05CD ATC Code	0.3	0.7829	0.069	0.1906
Hours of cognitive stimulation exercise/week	0.4681	2.866	0.079	0.1936
Diabetes mellitus	0.2143	1.374	0.079	0.1936
MEDAS-14	0.5284	5.143	0.119	0.2614
Depression according to GDS	0.169	1.169	0.119	0.2614
SCD: Language alteration time	0.4814	2.534	0.149	0.3112
Smoking	0.1422	1.753	0.168	0.3184
Hours of playing a musical instrument/week	0.1708	0.518	0.178	0.3184
Brain injury	0.1234	0.5463	0.188	0.3184
Adherence MEDAS	0.1341	0.8831	0.188	0.3184
Sex	0.1104	1.289	0.188	0.3184
Playing a musical instrument	0.07597	0.4145	0.218	0.355
Hours of physical exercise/week	0.1931	5.005	0.317	0.4979
Reading hours/week	0.08486	3.997	0.356	0.5373
Job	0.05875	3.92	0.366	0.5373
Number of alcohol units/week	0.08995	3.161	0.396	0.5621
HSV	0.04305	0.8968	0.426	0.5854
SCD: Time	−0.004185	5.444	0.535	0.7129
Number of cigarettes/day	−0.05327	1.002	0.555	0.7175
Type of pharmacy	−0.05896	2.41	0.574	0.7219
Depression	−0.05813	0.7875	0.663	0.7889
Depression treatment	−0.06122	0.6869	0.663	0.7889
Universal task	−0.02986	0.9747	0.693	0.8025
NSAIDs	−0.0684	0.7238	0.713	0.8043
BMI	−0.3249	7.664	0.782	0.8604
Alone	−0.1085	1.423	0.802	0.8607
BMI status	−0.2008	2.112	0.842	0.8713
Anticholinergic drugs	−0.1125	0.6182	0.852	0.8713
Hearing loss	−0.4794	1.166	0.980	0.9802

The columns are the name of the corresponding characteristics followed by two measures of importance: the corrected mean decrease in the corrected impurity (MDcI) and the mean decrease in the original Gini’s impurity decreased (MDI), the *p*-value by means of the Altmann’s method, and the false discovery rate (FDR). For the significance level, the following symbols are used: *p*-value < 0.01, * *p*-value < 0.05, ** *p*-value < 0.01, and SCD: Subjective Cognitive Decline; 5-GDS: 5-Point Geriatric Depression Scale; ATC: Anatomical Therapeutic Chemical classification system; MEDAS-14: 14-Point Mediterranean Diet Adherence Screen; HSV: Herpes Simplex Virus; NSAIDs: Nonsteroidal Anti-Inflammatory Drugs; BMI: Body Mass Index.

**Table A4:** Results of the tests of importance based on the decrease in the accuracy of the variables collected in ATENFARMA.

Characteristics	MDA	*p*-Value	FDR
Age	0.01197	0.010	0.1089109
Internet and social networks use	0.008851	0.010	0.1089109
Internet and social networks time	0.009246	0.010	0.1089109
Sleeping hours/day	0.005634	0.010	0.1089109
Stimulation exercise	0.002886	0.030	0.1867044
SCD: Object recognition time	0.004498	0.030	0.1867044
Type of pharmacy	0.002792	0.030	0.1867044
Diabetes mellitus	0.001466	0.040	0.2178218
Hours of cognitive stimulation exercise/week	0.002697	0.050	0.2420242
Family history of dementia	0.0009577	0.079	0.3168317
HSV	0.0007031	0.079	0.3168317
N05CD ATC code	0.0003047	0.119	0.3319189
Hypertension	0.001374	0.129	0.3319189
N05BA ATC code	0.0007797	0.129	0.3319189
Hypercholesterolemia treatment	0.00127	0.139	0.3319189
SCD: Language alteration time	0.0009499	0.149	0.3319189
Level of education	0.001908	0.149	0.3319189
Universal task	0.0006496	0.149	0.3319189
Sex	0.0007442	0.149	0.3319189
Brain injury	0.0002046	0.158	0.3319189
Job	0.002592	0.158	0.3319189
Chronic benzodiazepine use	0.0005939	0.178	0.3564356
Reading hours/week	0.001098	0.198	0.3788205
Smoking	0.0003714	0.248	0.4537954
Hours of physical exercise/week	0.0006644	0.297	0.5227723
Anticholinergic drugs	0.0001357	0.347	0.5864433
Depression according to GDS	0.0003289	0.376	0.6008877
Hours of playing a musical instrument/week	−6.2 × 10^−5^	0.386	0.6008877
Alone	5.729 × 10^−5^	0.396	0.6008877
Playing a musical instrument	−8.246 × 10^−5^	0.436	0.6204620
Adherence MEDAS	2.506 × 10^−5^	0.446	0.6204620
Other languages spoken	0.0001419	0.465	0.6204620
Number of cigarettes/day	−0.0001501	0.465	0.6204620
5-GDS	0.0004655	0.485	0.6278393
NSAIDs	−9.647 × 10^−5^	0.505	0.6347949
MEDAS-14	−0.0001613	0.535	0.6358041
SCD: Time	0.0006812	0.535	0.6358041
BMI	0.0006936	0.554	0.6420010
BMI status	0.0003043	0.663	0.7331562
Hearing loss	−0.0003603	0.673	0.7331562
Number of drugs	7.125 × 10^−5^	0.683	0.7331562
Depression	−0.0004091	0.842	0.8816596
Depression treatment	−0.0005473	0.901	0.9009901
Number of alcohol units/week	−0.001395	0.901	0.9009901

The columns are the name of the corresponding characteristics, the mean decrease in accuracy (MDA), the *p*-value by means of Altmann’s method, and the false discovery rate (FDR). For the significance level, 5-GDS: 5-Point Geriatric Depression Scale; MEDAS-14: 14-Point Mediterranean Diet Adherence Screen; HSV: Herpes Simplex Virus; SCD: Subjective Cognitive Decline; ATC: Anatomical Therapeutic Chemical classification system; NSAIDs: Nonsteroidal Anti-Inflammatory Drugs; BMI: Body Mass Index.

**Table A5:** Description of the variables in ATENFARMA.

Characteristic	Description
Age	Measured in years. All the patients are 50 years or older.
Cognitive decline	Being classified as having suspected cognitive decline due to testing positive in at least one of the MCI tests.
MIS	A value equal to or greater than 4 points is considered to indicate that the patient may be suffering from cognitive decline.
SPQMS	Three or more mistakes are considered to indicate that the patient may be suffering from cognitive decline.
SVF	If the patient enumerates fewer than 10 animals in 1 min, it is considered to indicate that the patient may be suffering from cognitive decline.
GDS-5	A score equal to or greater than 2 is considered to indicate that the patient may be suffering from depression.
MEDAS-14	A score lower than 7 points is considered to indicate low adherence to the Mediterranean diet.
Hearing loss	If the patient is suffering from hearing loss.
Hearing loss evolution (months)	For those patients suffering from hearing loss, how long have they been experiencing that loss? Measured in months.
Hearing aid use	For those patients suffering from hearing loss, are they using any devices to assist with their condition?
Chronic benzodiazepine use	Patients with chronic benzodiazepine use.
Type of benzodiazepine	ATC code.
Hypercholesterolemia	Patients diagnosed with hypercholesterolemia.
Hypercholesterolemia treatment	Those patients diagnosed with hypercholesterolemia adhered to treatment.
Hypercholesterolemia control	Those patients diagnosed with hypercholesterolemia and normal cholesterol values.
Depression	Patients diagnosed with depression.
Depression according to GDS	Patients with depression according to GDS-5.
Hours of physical exercise/week	How often does the patient engage in physical exercise.
Number of drugs	Prescribed medication regimen
Chronic NSAIDs use	Patients with chronic nonsteroidal anti-inflammatory drug use.
Depression treatment	Those patients adhered to depression treatment.
Family history of dementia	Having any direct family member with dementia.
Hypertension	Patients diagnosed with hypertension.
Hypertension treatment	Those patients diagnosed with hypertension adhered to treatment.
Hypertension control	Those patients diagnosed with hypertension and normal blood pressure values.
Stimulation exercise	Cognitive stimulation exercises to prevent cognitive decline.
Hours of cognitive stimulation exercise/week	How often does the patient engage in cognitive stimulation exercise.
BMI	Body Mass Index.
BMI status	Normal Weight: less than 25; Overweight: between 25 and 30; Obesity: greater than 30.
Obesity	Patients with a BMI indicative of obesity.
Overweight	Patients with a BMI indicative of overweight.
Reading hours/week	Number of hours reading per week.
Playing a musical instrument	Patients who play a musical instrument.
Hours of playing a musical instrument/week	How often does the patient play a musical instrument.
Adherence MEDAS	According to MEDAS-14, more than 2 points.
Job	Code assigned by the statistical agency in Spain based on the patient’s occupation.
Alone	Those patients living alone.
Loneliness feeling	Those patients living alone who feel loneliness.
Social contact	Those patients living alone who have frequent social contact.
Number of friends seen during the last week	For those living alone, how many friends have they visited in the last week.
SCD	Subjective Cognitive Decline.
SCD Time	Time suffering from subjective memory complaints.
SCD: Object recognition time	Time suffering from object recognition complaints.
SCD: Language alteration time	Time suffering from language alteration complaints.
Level of education	Reading/writing, primary, secondary, or higher education.
Other languages spoken	If they speak other languages.
Diabetes mellitus	Patients diagnosed with diabetes mellitus.
Diabetes mellitus treatment	Those patients diagnosed with diabetes mellitus adhered to treatment.
Diabetes mellitus control	Those patients diagnosed with diabetes mellitus and normal blood glucose values.
Smoking	If the patient is a smoker, a former smoker, or a non-smoker.
Number of cigarettes/day	For those who smoke, how many cigarettes do they smoke per day.
Number of years without smoking	For those who are former smokers, how long has it been since they quit smoking.
Number of alcohol units/week	How many alcohol units drunk per week.
Universal task	
HSV	Herpes virus.
HSV treatment	Those patients diagnosed with herpes virus adhered to treatment.
Internet and social networks use	If the patient uses the internet and social networks.
Internet and social networks time	For those using the internet and social media, how frequently do they use them?
Sex	Gender of the patient.
Sleeping hours/day	Number of hours sleeping a day.

MIS: Memory Impairment Screening; SVF: Semantic Verbal Fluency; SPMSQ: Short Portable Mental State Questionnaire; GDS-5: 5-Point Geriatric Depression Scale; MEDAS-14: 14-Point Mediterranean Diet Adherence Screen; NSAIDs: Nonsteroidal Anti-Inflammatory Drugs; BMI: Body Mass Index; SCD: Subjective Cognitive Decline; HSV: Herpes Simplex Virus.

## References

[1] Climent M.T., Molinero A. (2017). La farmacia comunitaria en la detección del deterioro cognitivo leve. Signos de alerta. Rev. Esp. Geriatr. Gerontol..

[2] Alzheimer’s Association (2019). Alzheimer’s Disease Facts and Figures. Alzheimers Dement..

[3] Duong S., Patel T., Chang F. (2017). Dementia: What pharmacists need to know. Can. Pharm. J..

[4] Mitchell A.J., Beaumont H., Ferguson D., Yadegarfar M., Stubbs B. (2014). Risk of dementia and mild cognitive impairment in older people with subjective memory complaints: Meta-analysis. Acta Psychiatr. Scand..

[5] Jessen F., Amariglio R.E., Buckley R.F., van der Flier W.M., Han Y., Molinuevo J.L., Rabin L., Rentz D.M., Rodriguez-Gomez O., Saykin A.J. (2020). The characterisation of subjective cognitive decline. Lancet Neurol..

[6] Jack C.R., Bennett D.A., Blennow K., Carrillo M.C., Dunn B., Haeberlein S.B., Holtzman D.M., Jagust W., Jessen F., Karlawish J. (2018). NIA-AA Research Framework: Toward a biological definition of Alzheimer’s disease. Alzheimer’s Dement..

[7] Bradford A., Kunik M.E., Schulz P., Williams S.P., Singh H. (2009). Missed and delayed diagnosis of dementia in primary care: Prevalence and contributing factors. Alzheimer Dis. Assoc. Disord..

[8] Borson S., Frank L., Bayley P.J., Boustani M., Dean M., Lin P., McCarten J.R., Morris J.C., Salmon D.P., Schmitt F.A. (2013). Improving dementia care: The role of screening and detection of cognitive impairment. Alzheimers Dement..

[9] Organización Mundial de la Salud, Organización Panamericana de la Salud (2013). Demencia: Una Prioridad de Salud Pública.

[10] Fogel H., Levy-Lamdan O., Zifman N., Hiller T., Efrati S., Suzin G., Hack D.C., Dolev I., Tanne D. (2021). Brain Network Integrity Changes in Subjective Cognitive Decline: A Possible Physiological Biomarker of Dementia. Front. Neurol..

[11] Climent M.T., Pardo J., Muñoz-Almaraz F.J., Guerrero M.D., Moreno L. (2018). Decision Tree for Early Detection of Cognitive Impairment by Community Pharmacists. Front. Pharmacol..

[12] Winblad B., Amouyel P., Andrieu S., Ballard C., Brayne C., Brodaty H., Cedazo-Minguez A., Dubois B., Edvardsson D., Feldman H. (2016). Defeating Alzheimer’s disease and other dementias: A priority for European science and society. Lancet Neurol..

[13] Livingston G., Huntley J., Sommerlad A., Ames D., Ballard C., Banerjee S., Brayne C., Burns A., Cohen-Mansfield J., Cooper C. (2020). Dementia prevention, intervention, and care: 2020 report of the Lancet Commission. Lancet.

[14] Rickles N.M., Skelton J.B., Davis J., Hopson J. (2014). Cognitive memory screening and referral program in community pharmacies in the United States. Int. J. Clin. Pharm..

[15] Moreno-Royo L., Climent M.T., Vilaplana A.M., Arnedo A., Vilar J. (2013). Estilos de vida asociados a deterioro cognitivo. Estudio preliminar desde la farmacia comunitaria [Life styles associated cognitive impairment. Study from the community pharmacy]. Rev. Investig. Clin..

[16] Murugesu K., Massé O., Maheu A., Guénette L. (2022). What is community pharmacists’ level of comfort and interest in managing patients with or at risk of major neurocognitive disorders?. Can. Pharm. J..

[17] Biecek P., Burzykowski T. (2021). Explanatory Model Analysis: Explore, Explain, and Examine Predictive Models.

[18] Murdaca G., Banchero S., Casciaro M., Tonacci A., Billeci L., Nencioni A., Pioggia G., Genovese S., Monacelli F., Gangemi S. (2022). Potential Predictors for Cognitive Decline in Vascular Dementia: A Machine Learning Analysis. Processes.

[19] Paluszynska A., Biecek P., Jiang Y. (2022). Explaining and Visualizing Random Forests in Terms of Variable Importance. R package version 0.10.1 [Internet]. https://CRAN.R-project.org/package=randomForestExplainer.

[20] Strobl C., Boulesteix A.L., Zeileis A., Hothorn T. (2007). Bias in random forest variable importance measures: Illustrations, sources and a solution. BMC Bioinform..

[21] Tricco A.C., Lillie E., Zarin W., O’Brien K.K., Colquhoun H., Levac D., Moher D., Peters M.D.J., Horsley T., Weeks L. (2018). PRISMA extension for scoping reviews (PRISMA-ScR): Checklist and explanation. Ann. Intern. Med..

[22] Levac D., Colquhoun H., O’Brien K.K. (2010). Scoping studies: Advancing the methodology. Implement. Sci..

[23] Faul F., Erdfelder E., Buchner A., Lang A.-G. (2009). Statistical power analyses using G*Power 3.1: Tests for correlation and regression analyses. Behav. Res. Methods.

[24] Böhm P., Peña-Casanova J., Gramunt N., Manero R.M., Terrón C., Quiñones-Ubeda S. (2005). Spanish version of the Memory Impairment Screen (MIS): Normative data and discriminant validity. Neurologia.

[25] López Pérez-Díaz A.G., Calero M.D., Navarro-González E. (2013). Predicción del deterioro cognitivo en ancianos mediante el análisis del rendimiento en fluidez verbal y en atención sostenida. Rev. Neurol..

[26] Price S.E., Kinsella G.J., Ong B., Storey E., Mullaly E., Pangnadasa-Fox L., Perre D. (2012). Semantic verbal fluency strategies in amnestic mild cognitive impairment. Neuropsychology.

[27] Martínez de la Iglesia J., Dueñas R.M., Onís M.C., Storey E., Mullaly E., Phillips M., Pangnadasa-Fox L., Perre D. (2001). Adaptación y validación al castellano del cuestionario de Pfeiffer (SPMSQ) para detectar la existencia de deterioro cognitivo en personas mayores de 65 años. Med. Clin..

[28] Pfeiffer E. (1975). A short portable mental status questionnaire for the assessment of organic brain deficit in elderly patients. J. Am. Geriatr. Soc..

[29] Olazarán J., Hoyos-Alonso M.C., del Ser T., Barral A.G., Conde-Sala J.L., Bermejo-Pareja F., López-Pousa S., Pérez-Martínez D., Villarejo-Galende A., Cacho J. (2016). Aplicación práctica de los test cognitivos breves. Neurol. íA.

[30] Ramos H., Moreno L., Gil M., García-Lluch G., Sendra-Lillo J., Alacreu M. (2021). Pharmacists’ Knowledge of Factors Associated with Dementia: The A-to-Z Dementia Knowledge List. Int. J. Environ. Res. Public Health.

[31] Schröder H., Fitó M., Estruch R., Martínez-González M.A., Corella D., Salas-Salvadó J., Lamuela-Raventós R., Ros E., Salaverría I., Fiol M. (2011). A Short Screener Is Valid for Assessing Mediterranean Diet Adherence among Older Spanish Men and Women. J. Nutr..

[32] Ros E. (2017). The PREDIMED study. Endocrinol. Diabetes Nutr..

[33] Ortega Orcos R., Salinero M.A., Kazemzadeh A., Aparicio S.V., Valle R. (2007). Validación de la versión española de 5 y 15 ítems de la Escala de Depresión Geriátrica en personas mayores en Atención Primaria. Rev. Clin. Esp..

[34] Wright M.N., Ziegler A. (2017). Ranger: A Fast Implementation of Random Forests for High Dimensional Data in C++ and, R.J. Stat. Softw..

[35] Breiman L. (2001). Random Forests. Mach. Learn.

[36] Nembrini S., König I.R., Wright M.N. (2018). The revival of the Gini importance?. Bioinformatics.

[37] Climent M.T., Ballesteros C., Colomer V., Botella P., Moreno L. (2015). Deterioro cognitivo y horas de sueño en mayores de 65 años no institucionalizados: Estudio en farmacia comunitaria. Farm. Comunitarios.

[38] Feijoo D., Ginesta E., Alambiaga-Caravaca A.M., Ruiz M.A., Ferrándiz E.C., Ripoll J.B., García M.A., Catalá M.T.C., Royo L.M. (2019). Potenciar la lectura desde la farmacia comunitaria en personas mayores para protegerlos del de- terioro cognitivo. Farm. Comunitarios.

[39] Ramos H., Alacreu M., Guerrero M.D., Sánchez R., Moreno L. (2021). Lifestyle Variables Such as Daily Internet Use, as Promising Protective Factors against Cognitive Impairment in Patients with Subjective Memory Complaints. Preliminary Results. J. Pers. Med..

[40] Ramos H., Pardo J., Sánchez R., Puchades E., Pérez-Tur J., Navarro A., Moreno L. (2021). Pharmacist-Physician Interprofessional Collaboration to Promote Early Detection of Cognitive Impairment: Increasing Diagnosis Rate. Front. Pharmacol..

[41] Mačeková Z., Krivošová M., Fazekaš T., Snopková M., Klimas J. (2022). Short cognitive screening in elderlies as a part of advanced pharmaceutical care in Slovak community pharmacies-The pilot study KOGNIMET-SK. Eur. Pharm. J..

[42] Ortiz L.A., García-Ribas G., Luna F.J., Álvarez M.P., Marcos N.S., López B.S. (2023). Usefulness of Community Pharmacy for Early Detection of Cognitive Impairment in Older People Using the IQ-CODE Questionnaire. J. Prev. Alzheimers Dis..

[43] Bragazzi N.L., Palombo-Ferretti F., Carbone R. (2023). Pharmacists-and Psychologists in Community Pharmacies-led Psychological Screening for Cognitive Issues in the Aftermath of the COVID-19 Pandemic” (PsyChOVID) WORKING GROUP. Community pharmacist- and psychologist-led program of neuropsychological screening in the aftermath of the COVID-19 pandemic: A cross-sectional survey. J. Health Soc. Sci..

[44] Macekova Z., Fazekas T., Krivosova M., Dragasek J., Zufkova V., Klimas J., Snopkova M. (2023). Identification of a Link between Suspected Metabolic Syndrome and Cognitive Impairment within Pharmaceutical Care in Adults over 75 Years of Age. Healthcare.

[45] Martínez L.A., García C., Moreno L. (2023). Estudio de los factores de riesgo de deterioro cognitivo en el medio rural: Metodología y pilotaje desde la farmacia comunitaria. Farm. Éuticos Comunitarios.

[46] Putignano S., Forgione L., Fusco M., Giacummo A., Magli E., Marino S., Marzano R., Putignano D., Santamaria F., Spatarella M. (2024). Early Detection Screening of Cognitive Decline in Patients Over 60 Years: ELDERCARE Study. J. Alzheimers Dis..

[47] García-Lluch G., Muedra-Moreno A., García-Zamora M., Gómez B., Sánchez-Roy R., Moreno L. (2024). Selecting a Brief Cognitive Screening Test Based on Patient Profile: It Is Never Too Early to Start. J. Clin. Med..

[48] Wolfsgruber S., Polcher A., Koppara A., Kleineidam L., Frölich L., Peters O., Hüll M., Rüther E., Wiltfang J., Maier W. (2017). Cerebrospinal Fluid Biomarkers and Clinical Progression in Patients with Subjective Cognitive Decline and Mild Cognitive Impairment. J. Alzheimers Dis..

[49] Borda M.G., Santacruz J.M., Aarsland D., Camargo-Casas S., Cano-Gutierrez C.A., Suárez-Monsalve S., Campos-Fajardo S., Pérez-Zepeda M.U. (2019). Association of Depressive Symptoms and Subjective Memory Complaints with the incidence of cognitive impairment in older adults with high blood pressure. Eur. Geriatr. Med..

[50] Numbers K., Crawford J.D., Kochan N.A., Draper B., Sachdev P.S., Brodaty H., Reddy H. (2020). Participant and informant memory-specific cognitive complaints predict future decline and incident dementia: Findings from the Sydney Memory and Ageing Study. PLoS ONE.

[51] Ababneh B.F., Ong S.C., Mahmoud F., Alsaloumi L., Hussain R. (2023). Attitudes, awareness, and perceptions of general public and pharmacists toward the extended community pharmacy services and drive-thru pharmacy services: A systematic review. J. Pharm. Policy Pract..

[52] Hussain R., Babar Z.U. (2023). Global landscape of community pharmacy services remuneration: A narrative synthesis of the literature. J. Pharm. Policy Pract..

[53] Nimpitakpong P., Chaiyakunapruk N., Dhippayom T. (2010). A national survey of training and smoking cessation services provided in community pharmacies in Thailand. J. Community Health.

[54] Scheffels J.F., Ballasch I., Scheichel N., Voracek M., Kalbe E., Kessler J. (2023). The Influence of Age, Gender and Education on Neuropsychological Test Scores: Updated Clinical Norms for Five Widely Used Cognitive Assessments. J. Clin. Med..

